# Prospective Large-Scale Field Study Generates Predictive Model Identifying Major Contributors to Colony Losses

**DOI:** 10.1371/journal.ppat.1004816

**Published:** 2015-04-13

**Authors:** Merav Gleit Kielmanowicz, Alex Inberg, Inbar Maayan Lerner, Yael Golani, Nicholas Brown, Catherine Louise Turner, Gerald J. R. Hayes, Joan M. Ballam

**Affiliations:** Monsanto Company, Chesterfield, Missouri, United States of America; Stanford University, UNITED STATES

## Abstract

Over the last decade, unusually high losses of colonies have been reported by beekeepers across the USA. Multiple factors such as *Varroa destructor*, bee viruses, *Nosema ceranae*, weather, beekeeping practices, nutrition, and pesticides have been shown to contribute to colony losses. Here we describe a large-scale controlled trial, in which different bee pathogens, bee population, and weather conditions across winter were monitored at three locations across the USA. In order to minimize influence of various known contributing factors and their interaction, the hives in the study were not treated with antibiotics or miticides. Additionally, the hives were kept at one location and were not exposed to potential stress factors associated with migration. Our results show that a linear association between load of viruses (DWV or IAPV) in *Varroa* and bees is present at high *Varroa* infestation levels (>3 mites per 100 bees). The collection of comprehensive data allowed us to draw a predictive model of colony losses and to show that *Varroa destructor*, along with bee viruses, mainly DWV replication, contributes to approximately 70% of colony losses. This correlation further supports the claim that insufficient control of the virus-vectoring *Varroa* mite would result in increased hive loss. The predictive model also indicates that a single factor may not be sufficient to trigger colony losses, whereas a combination of stressors appears to impact hive health.

## Author Summary

Roughly one third of the food supply relies on pollinating insects. The number of colony losses of the domesticated Honey Bee (*Apis mellifera*) has grown significantly in the past eight years, endangering pollination of crops like almonds. Recent research indicates that colony losses are influenced by a combination of several factors. We conducted an extensive and controlled study that allowed us to look at the contribution of different factors to colony loss. Results helped us build a predictive model showing that a single factor is often insufficient to trigger colony loss. Combination of stressors has shown to have greater impact on hive health; replication of the Deformed Wing Virus, stressful weather conditions, and *Varroa destructor* comprise the primary identified causes.

## Introduction

Pollination by wild or managed species of pollinators is essential to agricultural productivity. Honey Bees (*Apis mellifera*) play an essential role in this process by pollinating many important crops such as apples, almonds, and alfalfa [1,2]. In the United States (USA), almond-bearing acres have grown by 130% since 1982 and now rely on 1.6 million colonies (65%-70% of all USA colonies) to pollinate 740 thousand acres of almond trees [3].

In years prior to 2007, winter losses of hives averaged 10%–15%, represented by general decline in hive health, brood density, and total honeybee number. In 2007 beekeepers began to report unusually high losses of hives ranging from 30% to 90% [4]. The losses were associated with an unusual phenomenon of sudden disappearance of bees, with very few dead bees located near the colony. This phenomenon was designated Colony Collapse Disorder (CCD) [5,6]. Metagenomics analysis performed in 2007 identified the Israeli Acute Paralysis Virus (IAPV) as a potential cause of CCD [7,8], but further research showed that IAPV was present in the USA before the CCD epidemic [9]. Other research on CCD hives failed to show an association with IAPV [4]. Since 2007, colony losses have been monitored across the USA and found to average around 30% [10].

Recent research indicates that the decline of managed hives during winter months is influenced by a combination of several factors, including pests, parasites, bacteria, fungi, viruses, pesticides, nutrition, management practices, and environmental factors [4,11,12]. There is no consensus, however, regarding the relative importance of these factors, singly or in combination, in causing CCD [11].

Several studies have been performed in the USA and other world regions to identify the most significant factors associated with hive decline [4,7,13–18]. Most have focused on hive pathogens such as bee viruses, *Nosema*, and the *Varroa* mite. Cornman et al. performed a survey of pathogens in CCD and non-CCD hives, showing an increase of pathogens in collapsed hives; no association was determined for other factors such as weather, pests, or nutrition [17]. Runckel et al. performed a 10-month pathogen investigation using hives under migration stress and antibiotic treatment, and were able to show a correlation of hive collapse to various bee viruses and *Nosema* [16]. The ectoparasitic *Varroa destructor* mite, in combination with various bee viruses, is also associated with colony losses [7,19–26].

The *Varroa* mite is currently considered to be the most serious threat to honey bee populations worldwide [27]. *Varroa* has adapted to the developmental stages of the honey bee, entering uncapped brood cells to reproduce and feeding on the larval hemolymph after capping, causing nutrient depletion and weakening the larvae. Several bee viruses have been reported to be transmitted by and replicated in *Varroa* mites who act as an alternative host. These include Deformed Wing Virus (DWV), Kashmir Bar Virus (KBV), Sacbrood Virus (SBV), Acute Bee Paralysis Virus (ABPV), and Israeli Acute Paralysis Virus (IAPV) [24,28–32]. Surveys monitoring virus and *Varroa* mite levels have been supplemented by modeling approaches that are found to predict and understand the dynamics of the *Varroa*-virus interaction in the hive and their effect on hive health [33–35].

Beekeepers monitor *Varroa* mite levels extensively and use several acaricides to maintain low *Varroa* levels. However, determining presence of pathogenic bee viruses and whether they replicate is complex and not always available to beekeepers. Furthermore, not all bees exhibit a phenotype when virally infected, thus complicating any diagnosis and making prediction of viral outbreaks even more difficult.

To further dissect factors that influence hive health, we conducted large-scale controlled winter trials at three locations across the USA. Each site contained approximately 60 monitored hives that were not treated with antibiotics or acaricides in order to better understand the effect of *Varroa destructor*, bee viruses, and *Nosema* on hive health. The trials were conducted without exposing hives to migratory stress. A weather station was placed at each location for daily monitoring of temperature, humidity, and precipitation. The hives were assessed four times during a period of 7 months. The assessment included *Varroa* levels, prevalence and replication of 8 bee viruses, *Nosema ceranae* levels, hive strength, measured by the Almond Grower Method (AGM), and total adult bee number.

## Results

### Trial design and assessment of hive strength

**Fig 1:**
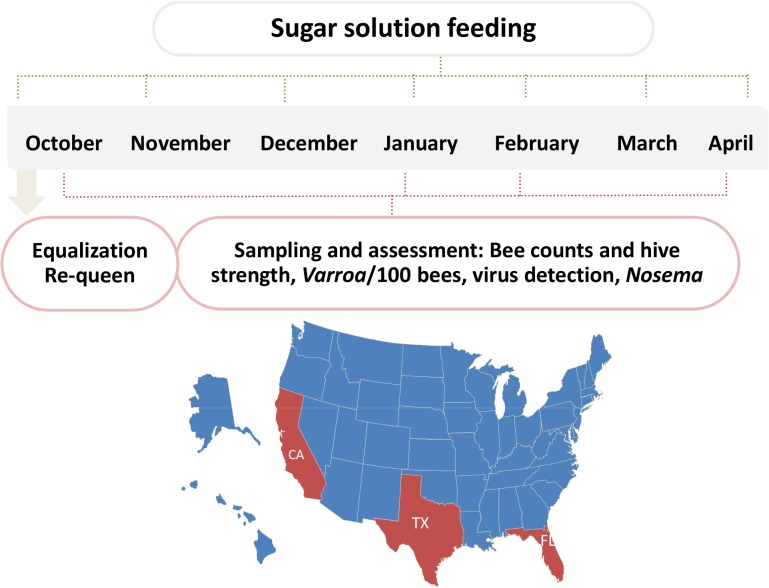
Schematic illustration of trial schedule and map. 60 hives in each site were requeened and equalized to have 7 bee frames and 3–4 brood frames. Hives were fed once a month with 60% sugar syrup and sampled four times during the trial for hive strength, bee number, bee viruses, *Nosema*, *Varroa* counts.

**Fig 2:**
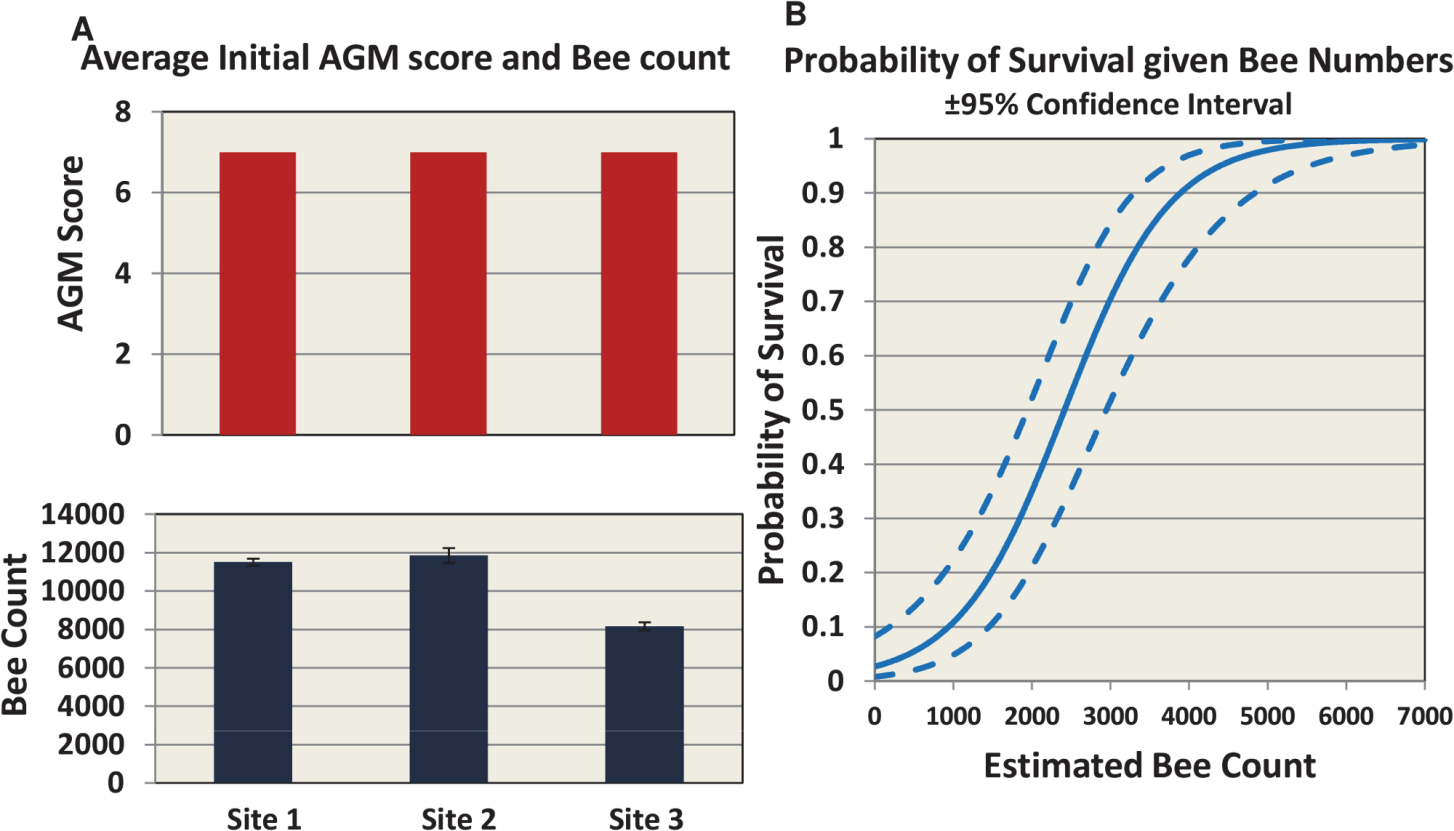
Initial AGM and bee counts and survival given hive population. **(A**) hives were assessed using Almond Grower Method (top panel) and bee number (bottom panel, SE) at each site. (**B**) Probability of hive survival according to bee number.

The trial was carried out in 3 different locations across the US to represent 3 different climate and geographical conditions: mountain area of Northern California (site 1), costal area of Florida (site 2) and Southeast Texas (site 3) (Fig 1). Hives were internally fed monthly with sugar syrup and were assessed four times for hive strength, virus levels, *Nosema ceranae*, and *Varroa* mite counts (Fig 1). Two methods were used to assess hive strength: the Almond Grower Method (AGM), used by beekeepers to assess hive strength as number of bee covered frames before almond pollination; and imaging software, counting bees from frame images [36]. The number of bees in the hive provides a reliable proxy to the comparative strength of the hive. Fig 2A shows different results between the two methods; while the AGM method showed equal hive strength at start point in all three sites, the frame imaging method indicates that Site 3 had significantly (P<0.05) fewer bees than the other two sites.

To allow for consistency between sites, different inspectors and beekeepers, we defined in the trial protocol a collapsed hive as a hive with AGM assessment of less than or equal to 1 bee frame coverage. In our study the probability of overwinter survival for a hive, based on bee population, is estimated to be 50% when bee number drops below 2500 and 90% when at least 4000 bees are present (Fig 2B).

### Pathogen prevalence

**Fig 3:**
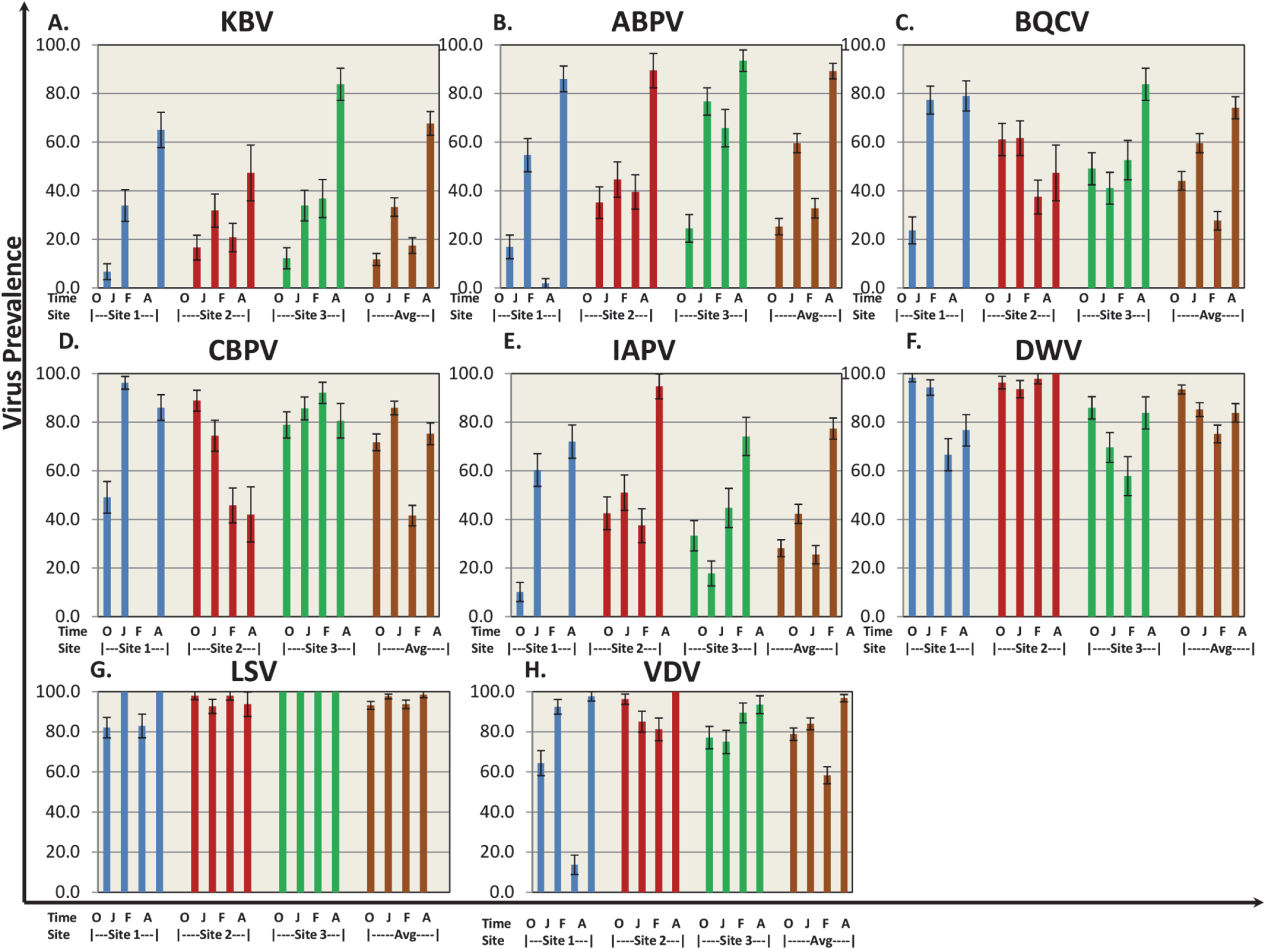
Virus prevalence by location and collection time. 8 bee viruses (**A)** Kashmir Bar Virus (KBV). (**B)** Acute Bee Paralysis Virus (ABPV). (**C)** Black Queen Cell Virus (BQCV). (**D)** Chronic Bee Paralysis Virus (CBPV). (**E)** Israeli Acute Paralysis Virus (IAPV). (**F)** Deformed Wing Virus (DWV). (**G)** Lake Sinai Virus (LSV). (**H)** *Varroa* Destructor Virus–1 (VDV-1)) were identified using QuantiGene Plex 2.0 RNA assay (Affymetrix). The prevalence was determined as % of sampled hives at each site. O—October, J—January, F—February, A—April. Standard error is indicated.

Eight bee viruses were assessed for prevalence (defined as percentage of hives where the virus was detected) throughout the trial using QuantiGene Plex 2.0 platform. Bee virus prevalence reported here is a snapshot of the prevalence for those hives that were classified as live at the sampling time. As the study progressed, the number of sampled hives decreased due to hive loss. The temporal patterns in virus prevalence were similar for the subset of hives that had measurements at every sampling period and those reported here for all live hives at each sampling period (S1 Table). KBV, ABPV, and IAPV exhibited similar patterns, starting with relatively low levels at the beginning of the trial (October time point Fig 3A, 3B, and 3E) and increasing by trial end to >65% across sites. DWV was found at high prevalence (75%-95%) throughout the trial with no significant difference among sites. BQCV and CBPV ranged between 25%-95% prevalence across sites throughout the trial. Lake Sinai Virus (LSV) was present in >90% of the hives across sites and time points. One striking difference was found at Site 1, where prevalence of paralysis viruses (KBV, ABPV, BQCV, CBPV, IAPV) dropped dramatically in the February assessment, while DWV and LSV prevalence remained high.

*Nosema ceranae* was analyzed by QuantiGene Plex 2.0 platform using two different probes to verify consistency of results. *Nosema* prevalence averaged 60–85% across sampling times (Table 1), but differences in prevalence were noted among sites at different sampling times.

**Fig 4:**
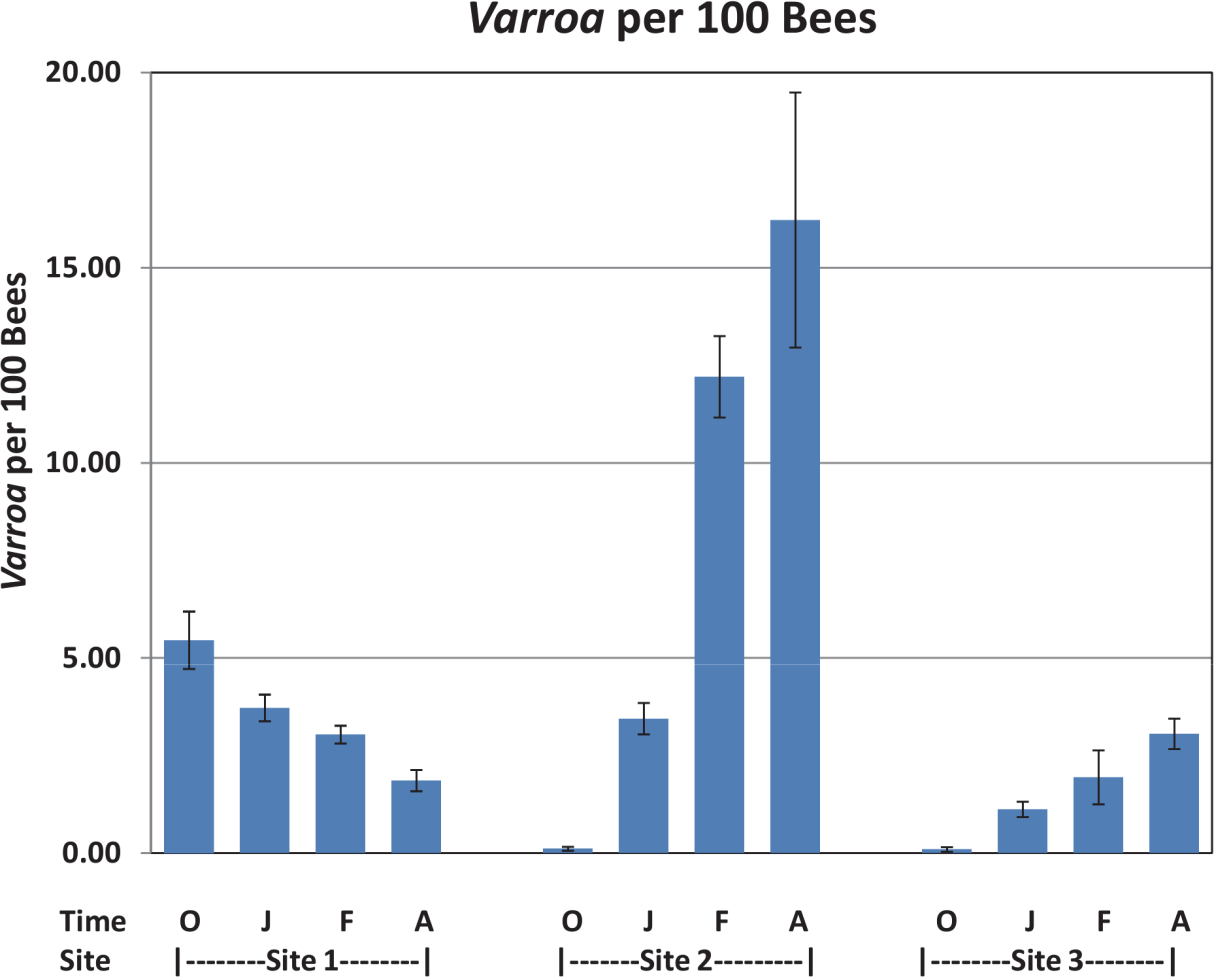
*Varroa* per 100 bees by location and collection time. *Varroa* counts per 100 bees determined at all locations for all collection time points. Results are average *Varroa* counts per site per assessment time. O—October, J—January, F—February, A—April. Standard error is indicated.

Significant increases in *Varroa* counts per 100 bees were observed across time at sites 2 and 3 while a significant decrease was found at Site 1 (Fig 4).

**Table 1:** Nosema prevalence by location and collection time.

		Percent of Hives with *Nosema* ceranae			
	Time	Site 1	Site 2	Site 3	Avg
*Nosema* ceranae	October	33.9	81.5	78.9	64.1
	January	88.7	83.0	48.2	72.4
	February	88.2	62.5	63.2	72.3
	April	86.0	68.4	93.5	84.9

*Nosema* was determined by QuantiGene Plex 2.0 RNA assay (Affymetrix). The prevalence was determined as percentage of hives with positive *Nosema* values out of the sampled hives.

### Virus levels in *Varroa* mites and bees

**Fig 5:**
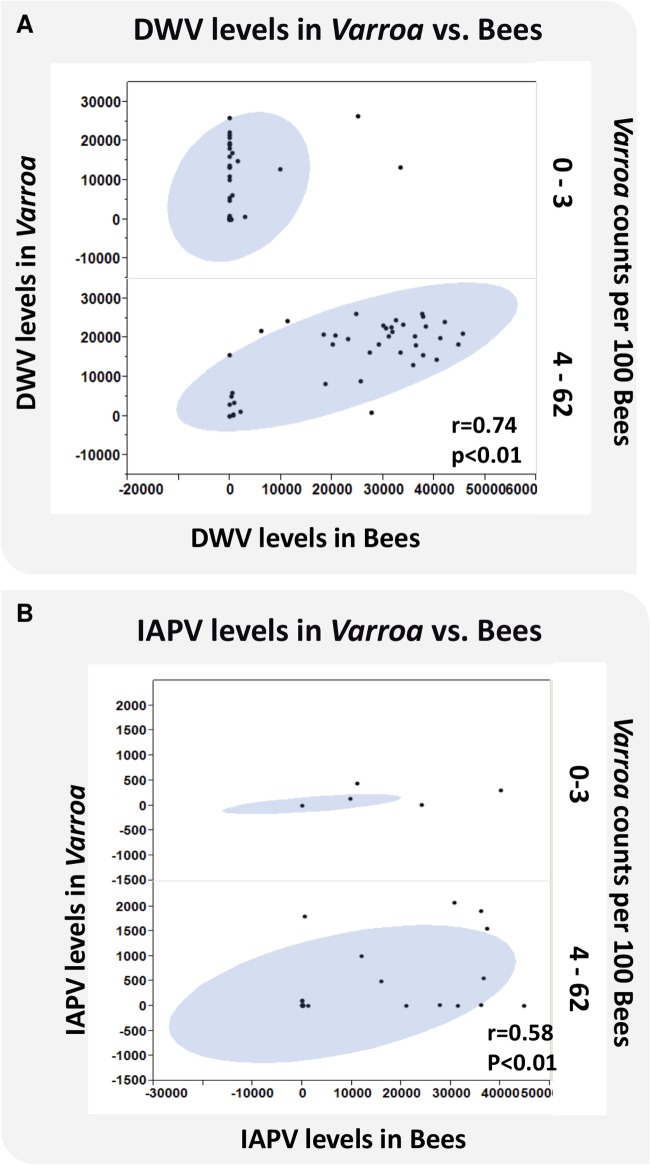
Virus levels of DWV and IAPV in bees and *Varroa* and the correlation to *Varroa* counts. DWV and IAPV were detected in bees and *Varroa* mites sampled from each hive using QuantiGene Plex 2.0 RNA assay (Affymetrix). (**A)** X axis represents the levels of DWV in bees. Y axis represents the levels of DWV in *Varroa*. Z axis represents *Varroa* mite count per 100 bees. (**B)** X axis represents the levels of IAPV in bees. Y axis represents the levels of IAPV in *Varroa*. Z axis represents *Varroa* mite count per 100 bees. The oval represents coverage of 95% of the data.

The relationship between DWV and IAPV virus levels in bees and *Varroa* infestation, defined as the number of phoretic mites per 100 bees, is depicted in Fig 5A and 5B. Results show a significant linear association of virus levels of the two viruses in bees and *Varroa* at high *Varroa* infestation (>3 *Varroa* mites per 100 bees). At low *Varroa* infestation (≤3 *Varroa* mites per 100 bees), there were insufficient numbers of colonies with non-zero levels of DWV and IAPV in bees to determine a correlation. DWV can be found at high virus levels in *Varroa*, but not in bees at low *Varroa* infestation (Fig 5A).

### Relationships between *Varroa* count and pathogen load

Two sites exhibited a positive linear relationship between *Varroa* mite infestation and levels of at least one virus. *Varroa* infestation was positively associated with DWV (Sites 1 and 2), IAPV (Site 2), and VDV (Site 2) (Table 2). No significant association between *Varroa* infestation and viral load was found at Site 3. The variation in *Varroa* mite number was narrower at Site 3 than Sites 1 and 2, which may have limited the ability to detect significant relationships. Positive linear relationships were found between *Nosema ceranae load* and levels of the Dicistroviridae family of viruses (ABPV, BQCV, CBPV, IAPV, KBV) and LSV at all sites. Associations with DWV or VDV-1 were less consistent (Table 2).

**Table 2:** Linear relationship between *Varroa* counts, Nosema Ceranae and bee viruses after accounting for trends over time.

**Change in**	**Location**	**Number of hives**								
			**DWV**	**VDV**	**ABPV**	**BQCV**	**CBPV**	**IAPV**	**KBV**	**LSV**
***Varroa* mite**	**Site 1**	**58**	**++**							
	**Site 2**	**60**	**+++**	**+++**				**++**		
	**Site 3**	**60**			**-**				**-**	
***Nosema* ceranae**	**Site1**	**59**		**+**	**+++**	**+++**	**+++**	**+++**	**++**	**+**
	**Site 2**	**60**		**+**	**+++**	**+++**	**+++**		**+++**	**+**
	**Site 3**	**60**	**++**	**+++**	**+++**	**+++**	**+++**	**+++**	**+++**	**+++**

Plus indicates positive relationship, minus indicates negative relationship.

+++ or—P-value <0.01; ++ or—P-value <0.05; + or—P-value <0.10

Replication of DWV, but not IAPV or LSV, increased significantly with an increase in *Varroa* infestation at two sites (Table 3). Increase in *Nosema* was significantly associated with increase in replicating IAPV at 3 sites, with DWV at 2 sites and with LSV at 1 site (Table 3). Data were not sufficient to determine association between replication of ABPV or KBV with *Varroa* mite increase or *Nosema ceranae*.

**Table 3:** Linear relationship between *Varroa* or *Nosema* and replication (RC) of IAPV, DWV, ABPV, KBV after accounting for trends over time.

	**Location**	**DWV-RC**	**IAPV-RC**	**LSV-RC**	**ABPV-RC**	**KBV-RC**
***Varroa* mite**	**Site 1**	**++**			**—**	
	**Site 2**	**++**			**IS**	**IS**
	**Site 3**				**IS**	**IS**
***Nosema* ceranae**	**Site 1**	**+++**	**+++**			
	**Site 2**	**+**	**+++**		**IS**	**IS**
	**Site 3**	**+++**	**+++**	**+++**	**IS**	**IS**

Plus indicates positive relationship, minus indicates negative relationship.

+++ or—P-value <0.01; ++ or—P-value <0.05; + or—P-value <0.10 IS—Insufficient Data

### *Varroa destructor*, viruses, and colony losses

Table 4 shows the colony loss pattern throughout the trial. During the January and February assessments, all sites exhibited similar losses of about ≃22%; while in April there was an increase in colony losses at site 2 and 3 compared to previous months, and in comparison to site 1(30 colonies at Site 2 and 13 colonies at Site 3). Temperature loggers at each site indicated that, while at Site 1 minimum temperatures remained below 6°C throughout the trial, Sites 2 and 3 showed average temperature increases to above 7°C in April (Table 5).

**Table 4:** Number of collapsed hives (hives with AGM≤1) per assessment time.

Month of assessment	Number of collapsed hives		
	Site 1	Site 2	Site 3
October	0	0	0
January	1	2	7
February	13	10	6
April	**3**	**30**	**13**
Total	17	42	26
**Percent of losses**	**28.8%**	**70.0%**	**43.3%**

Table 4 represents number of collapsed colonies out of 60 monitored colonies at sites 2 and 3 and 59 monitored colonies at site 1.

**Table 5:** Average minimum and maximum temperature (°C) across sites during the trial.

	**Site 1**		**Site 2**		**Site 3**	
**Temperature (°C)**	**min**	**max**	**min**	**max**	**min**	**max**
November	5	15	10	26	10	26
December	2	11	10	26	10	22
January	0	13	12	28	8	19
February	-1	16	11	28	9	21
March	4	19	7	26	10	23
April	6	22	17	32	13	25

Temperatures were measured by a weather monitoring station at each site.

**Fig 6:**
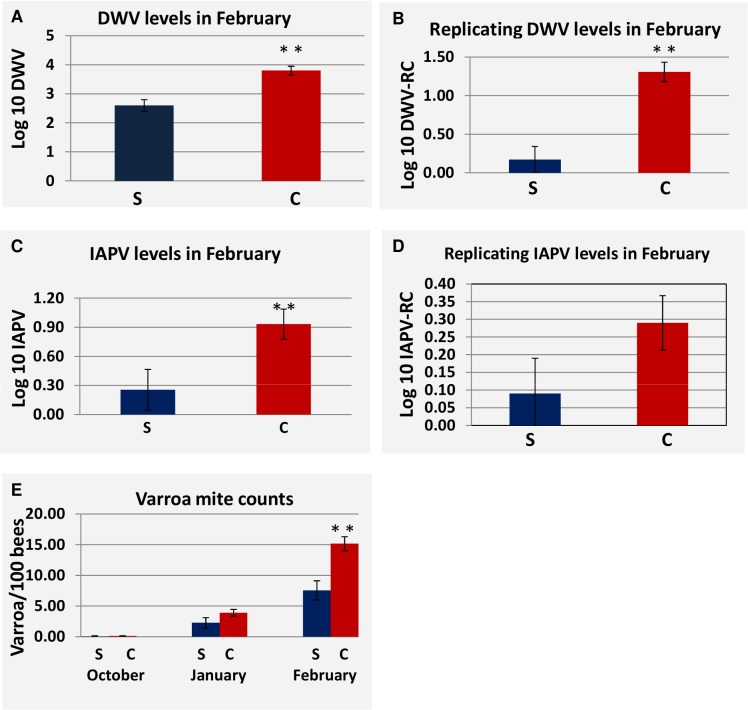
Difference in viral load, viral replication and *Varroa* counts as measured in survived hives and collapsed hives at site 2. (S) Represents hives that survived through the April assessment, (C) represents hives that Collapsed by the April assessment. P-value < 0.05 is indicated as (**). (**A)** DWV load at the February assessment for survived vs. collapsed hives. (**B)** Replication of DWV at the February assessment for survived hives vs. collapsed hives. (**C)** IAPV load in at the February assessment for survived vs. collapsed hives. (**D)** Replication of IAPV at the February assessment for survived hives vs. collapsed hives. (**E)** Comparison of *Varroa* counts per 100 bees in survived vs. collapsed hives across the trial. Standard Error is indicated.

At site 1 replicating DWV was greater in collapsed hives in the October measurement but not in January or February (S2 Table). At Site 2, DWV load was consistently greater in collapsed hives and significantly so (P<0.05) at the February collection period after which the greatest loss in hives occurred (Fig 6A). IAPV and replication of DWV were also significantly greater at the February collection (Fig 6B and 6C). Although not significant, we found that the replication of IAPV was higher in collapsed hives (P<0.15 Fig 6D). At Site 3, DWV and IAPV loads were significantly greater in collapsed hives in January (S2 Table). Replication of IAPV, however, was found to be consistently greater in survived hives and significantly greater at the February time period (S2 Table).

*Varroa* mite counts increased throughout the trial at Site 2 and differed significantly between collapsed and survived hives in February (Fig 6E), where levels of *Varroa* mites in collapsed hives averaged 15 mites per 100 bees. A similar pattern of increasing *Varroa* infestation over time with greater levels in collapsed hives and significantly so at the February collection was noted at Site 3 but not at Site 1.

### Predictive modeling of colony losses

**Fig 7:**
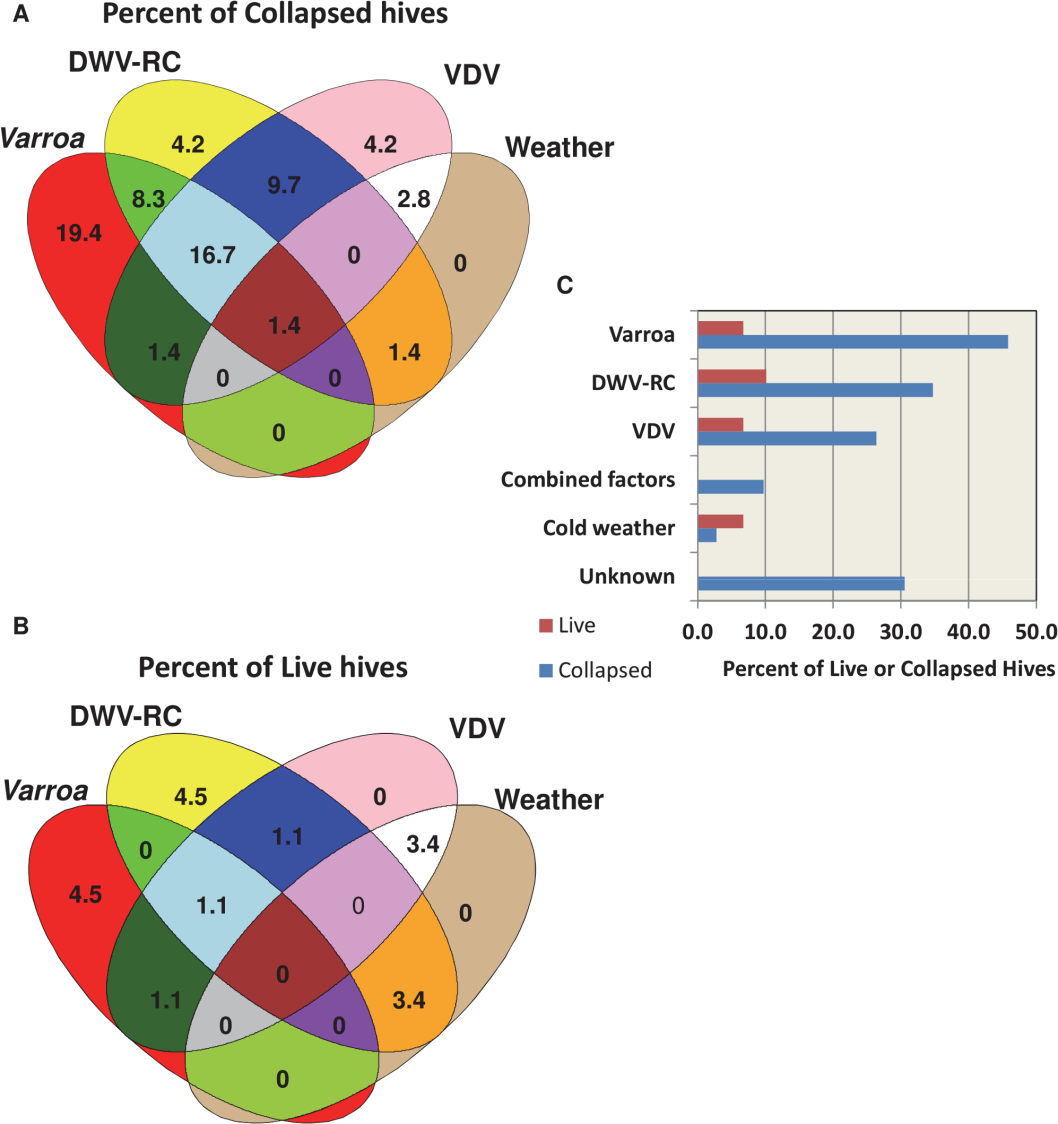
Relative contribution of different factors to hive collapse. **(A)** Schematic illustration of relative contribution of different factors (*varroa* mite, DWV-RC, VDV and extreme weather conditions) to colony losses **(B)** Schematic illustration of contribution of different factors to the predicted collapse of surviving hives. **(C)** Percent contribution of factors to the actual collapse and predicted collapse of hives.

The predictor variables in the final logistic model were DWV replication, VDV load, average 10-day minimum temperature, average maximum temperature, IAPV replication, and location. A consistent linear association was found between collapsed hives and DWV replication. At DWV replication levels of >32 QG units, 80% of the hives collapsed. In the predictive model, increased DWV replication, VDV load, sustained cold temperatures, and sustained warm temperatures were found to increase the probability of colony losses. A counter-intuitive association between high IAPV replication and hive survival was found. Further examination of this factor revealed 10 surviving hives at Site 3 with high levels of IAPV replication and DWV replication. Replicating IAPV helped to correctly predict survival of those hives. At the other two sites, high levels of IAPV replication were associated with colony losses. *Nosema ceranae* was not chosen as a predictive marker by the model. *Varroa* also was not selected as a predictor due to lack of a linear association across all levels of *Varroa* infestation. However, at loads ≥8 *Varroa*/100 bees, 87% of the hives collapsed. The relative contribution of each factor to colony losses is depicted in Fig 7. Although a linear relationship between collapse and *Varroa* load was not found, *Varroa* is included in the figure to account for collapse in hives with high infestation. Collapse was attributed to *Varroa* at ≥8 mites/100 bees, to DWV replication at ≥32 QG units and to VDV at ≥300 QG units. Collapse of hives that were correctly predicted as collapsed but did not meet any individual threshold level was attributed to a combination of factors at lower threshold levels. Cold weather in combination with at least one other factor, but not by itself, was included as contributing to collapse. Warm weather was associated with increased *Varroa*, DWV replication or VDV levels and was not considered as a direct factor in collapse. *Varroa* infestation, DWV replication, VDV loads, and cold weather accounted for 69% of the collapsed hives. The reason for collapse in the remaining 31% is unknown. The same criteria, when applied to the surviving hives, predicted 19% should have collapsed.

## Discussion

In the last seven years, mean annual colony losses across the USA increased to approximately 30%. Extensive research has been performed in the previous decade to characterize reasons for colony losses [4,6,7,13,17,37–39].

Our study, a comprehensive, controlled experiment with a large number of monitored hives (179 hives in three locations), examines hive losses as a multi-factorial event. Evaluated factors include *Varroa* mite, bee viruses, *Nosema ceranae*, weather, and location, but do not account for factors such as migration, treatment of antibiotics and mitocides, nutrition or exposure to pesticides.

### Pathogen prevalence changes between sampling time points, but not between sites

Several pathogens, such as bee viruses, *Nosema ceranae* and *Varroa destructor* have been proposed as contributing to increased winter losses of bees [15,37,40–43].

In 2012, the National Honey Bee Pests and Diseases Survey Report published the prevalence of bee viruses *Nosema* and *Varroa destructor* across the USA between the years 2009–2011 [18]. Similar levels of bee viruses, with slight differences among sites, are reported in this study (Fig 3). The 2012 Survey Report found that IAPV prevalence varies from year to year and increases between January and April. A similar increase from January to April, with higher levels of IAPV, KBV, ABPV, and BQCV, was reported here. LSV was surveyed by Runckel at al. showing a peak in prevalence in January, whereas here we show high virus prevalence throughout the trial [16]. Difference in detection methods (QuantiGene Plex 2.0 platform vs. QPCR), year of sampling, or the fact that the hives in this study were not treated could account for differences noted between the two studies. Francis et al. compared IAPV, KBV, and ABPV viral levels in their study in mitocide-treated and untreated hives and showed an increase in viral load in the untreated groups [43]. This increase could be correlated to an increase in *Varroa* mite infestation reported in our study (Fig 4), as the mite was found to transmit these viruses to bees [21,29–31,43]. Prevalence of these viruses exhibited reduction at the February time point at Site 1, potentially caused by virus-carrying bee mortality between the January and February time points. Unlike Cornman et al., in our study, the overall picture of virus prevalence was similar across all sites and no difference was found between the different regions in the USA [17].

The microsporidia *Nosema ceranae* is associated with colony losses, especially in Europe [44,45]. Botias et al. showed presence of *Nosema* spores in honey and honey bee samples taken as early as 2000, and an increase in prevalence from 30% in 2002 to 47% in 2007 [46]. Our data from 2012/2013 show the prevalence of *Nosema* to be 60–80%, depending on the site and month of sampling (Table 1). Similar data was reported by others [17,18]. Runckel et al. reported lower levels of *Nosema*, but in their study, hives were treated with fumagillin [16].

*Varroa destructor* is considered to be one of the main causes of hive decline; therefore, we monitored its levels (expressed as number of mites per 100 bees) throughout the trial. Rennich et al. reported *Varroa* mite levels of 2–8 per 100 bees depending on the season and year of sample [18]. The average mite number in their survey is only from hives where *Varroa* was found, whereas we show an average count of all hives (Fig 4). Sites 2 and 3 started with very low levels of *Varroa*, as the participating beekeepers treated against *Varroa* prior to start point. Mite levels at these sites increased during the trial, averaging as high as 15 mites per 100 bees at Site 2. Unlike Sites 1 and 3, the temperature at Site 2 (Table 5) was high throughout the trial. Bee number of survived hives (S3 Table) at Site 2 did not decrease for the first 5 monitored months, indicating the presence of brood throughout the trial, hence the opportunity for *Varroa* mite to propagate. Sites 1 and 3 experienced a roughly 50% drop in the bee population during the first 5 months of our study (S3 Table), most likely due to low winter temperatures (Table 4), thus preventing bee brood development as well as *Varroa* reproduction.

### Association between Viral load in *Varroa* and bees is found at high mite infestation

Previous studies have demonstrated the correlation between *Varroa* mite infestation and viruses, especially DWV [21,24,28–31], the association between viral replication in mites and development of crippled wings [19,24,47,48], and the correlation between DWV viral copies in bees and mite infestation [43].

This study supported a direct association between 3 parameters: viral load (DWV or IAPV) in bees, viral load in mites, and mite infestation level. Given the observational nature of this study, the reported associations do not imply causation. The DWV viral load in bees can remain low in the presence of a high DWV copy number in *Varroa* as long as *Varroa* infection is low (≤3 mites/100 bees). At mite levels >3 mites/100 bees, a linear association can be found between the DWV and IAPV loads in the mite and in the bees. Martin et al. showed in their survey that, once *Varroa* penetrated the Hawaiian islands, DWV increased both in prevalence and in copy number [24]. Similarly, Mondet et al. showed that presence of DWV or KBV in bees correlates to their presence in the mites [49]. The penetration of *Varroa* mite to New Zealand also increased the number of different bee viruses in infested hives as compared to hives from *Varroa*-free areas. These results support the theory of direct transmission of bee viruses between *Varroa* to bees.

To further characterize the relationship between *Varroa* infestation and different bee viruses, the association between *Varroa* mite infestation and viral load and replication was tested at each site. At Site 1, only DWV was positively correlated with mite infestation, while at Site 2, DWV, VDV-1, and IAPV were positively associated. At site 3, non-significant negative correlations were found between mite infestation, KBV and ABPV. Similar negative correlation was reported by Mondet et al. once *Varroa* penetrated New Zealand [49]. They also showed that different viruses can be found in the bees in correlation to the number of years *Varroa* infested the island. The differences between sites could also result from different viruses being carried by *Varroa* at different locations as reported by others [24,30,31]. DWV replication increased proportionately to mite infestation, but the replication of IAPV and LSV did not associate with mite infestation. Sumpter et al. have used a mathematical model to investigate the relationship between mite load and viral epidemic potential within a colony, demonstrating that, because the paralysis viruses (IAPV/ABPV/KBV) are harmful to bees at low levels, a pupa infected in a brood cell by a mite with APV will die before reaching adulthood [35]. Pupal mortality indicates that the virus will not spread in the hive unless transmitted by the *Varroa* to adult bees. DWV is less virulent in its effects and infected pupae most probably will emerge with the virus allowing the epidemiological correlation of replicating virus with *Varroa* infestation [35].

### Increase in viral load correlates with increase in *Nosema ceranae*

Our study shows that increase in *Nosema ceranae* is associated with an increase of all tested paralysis viruses, while having low correlation, if any, with DWV, VDV-1, or LSV. These results are supported by data obtained by Martin at al., in which no correlation was found between *Nosema ceranae* and DWV viral load [50]. The association between the microsporidia to the paralysis viruses has been demonstrated; Toplak et al. showed potential synergistic effects when co-infecting bees with CBPV and *Nosema ceranae*, yet this association does not necessarily imply direct causation and could result from effects of high *Nosema* or viral load on the immune system of the bee [51]. Antunez et al. showed that infection of *Nosema ceranae* leads to suppression of bee immune response, which could lead to an increase in bee pathogens. [52].

### Prediction model indicates that combination of *Varroa destructor* and replication of DWV are the major markers of colony losses

Suggested reasons for hive decline have been numerous: *Varroa* mite, bee viruses, *Nosema*, nutrition, extreme cold, beekeeping practices, and pesticides. This study addressed colony loss as a multi-factorial problem and identified DWV replication, *Varroa* infestation and VDV loads as influential in colony losses. Weather could not be considered as a stand alone factor for collapse since all hives at each location were exposed to the same weather patterns. There were, however, notable differences in weather patterns among sites and the predictive model found both sustained cold and sustained warm weather to increase the probability of colony collapse. A sustained cold period at site 1 was predicted to influence the February colony loss. The sustained warm weather at site 2 was accompanied by increased levels of at least one other risk factor. Late colony collapse at this site was attributed to high levels of *Varroa* infestation, DWV replication or VDV which may have increased under mild winter weather conditions.

Factors contributing to colony losses were *Varroa* infestation (≥8/100 bees), DWV replication (≥32 QG units), VDV-1 load (≥300 QG units), combinations of *Varroa* infestation, DWV replication and VDV-1 at lesser threshold levels, and cold weather in combination with at least one other contributing factor (Fig 7). The model suggests that non-treated hives with increased mite populations are more likely to decline due to mite infestation, as reported by others [37,42]. Based on the factors measured in this study, DWV replication has the greatest impact on colony loss in treated hives where mite population is controlled. Interestingly, the model predicted that a portion of the surviving hives had a greater than 50% probability of collapse. These hives were largely subject to high levels of single risk factors; further suggesting collapse is enhanced by the presence of multiple factors.

Our study supports the hypothesis that combinations of factors contribute to colony losses. With no available treatment against *Varroa*, its levels can exceed 8 mites per 100 bees, causing hives to collapse. At lower mite infestation rates, replication of bee viruses takes an active role in the collapse. According to our model, approximately 70% of hive collapse is caused by *Varroa* and bee viruses. Control of mite and viral levels may mitigate colony loss, resulting in levels more acceptable to the apiary industry.

## Materials and Methods

### Hive equalization

Three commercial beekeepers participated in the trial. At each site hives were re-queened with queens of the same age and genetic background, and equalized to have 7 frames covered with bees and 3–4 frames covered with capped brood. Queens were purchased from Kona Queen Hawaii, Inc.; at study initiation, queen acceptance was verified.

### Bee collection

Bees intended for Quantigene Plex 2.0 assay and *Nosema* counts were collected from the outer frame in a 50mL tube. Immediately following collection, samples were placed on dry ice and were kept at -80°C until analyzed. For *Varroa* counts, half a cup of bees was sampled from the inner frame into Wide-Mouth HDPE Packaging Bottles with PP closure (Thermo Scientific cat 03-313-15D), which contained 70% alcohol solution.

### *Varroa* mite collection

To collect *Varroa* mites for virus analysis, the sugar shake method, used by beekeepers to monitor mite level or as treatment against *Varroa*, was used. A cup of powdered sugar was placed on top of the frames and spread using a hive tool or a paint brush, allowing adult bees to be covered with sugar powder. The sugar powder causes the majority of mites to dislodge from their host and fall down onto the bottom board. A white paper was placed on a bottom board of each hive. After five minutes the bottom board was removed and live *Varroa* mites were collected into 50mL tubes using a paint brush. The mites were immediately placed on dry ice and kept at -80°C until analyzed.

### Almond grower assessment method

Almond grower assessment method (AGM) was performed by the beekeepers or the assigned study monitor that was trained to perform the assessment. Hives were opened and graded by the number of covered bee frames assessed after looking at the top and bottom of each hive.

### Weather collection data

A weather collection station monitoring temperature, humidity, and precipitation was placed at each site (Phytech, ILS). The data was transmitted in real-time over a cellular network and collected in our computers.

### Bee counts

Frames with bees were slowly removed to avoid disruption and placed on a frame holder. Photos were taken from both sides of the frame. Total number of bees on each frame was determined using image recognition software (IndiCounter, WSC Regexperts).

### Quantigene assay

Quantigene, a quantitative, non-amplification-based nucleic acid detection analysis, was performed on total lysate from frozen honey bees or *Varroa* mite samples. The oligonucleotide probes used for the QuantiGene Plex 2.0 assay were designed and supplied by Affymetrix, using the sense strand of bee virus sequences as template or negative strand for replicating virus. The probe, designed to detect the sense strand, reflects the presence of virus (viral load) and probe designed to detect the anti-sense strand reflects level of viral replication. Housekeeping gene probes were designed from sequences of *Apis mellifera mellifera* Actin, Ribosomal protein subunit 5 (RPS5), and Ribosomal protein 49 (RP49). For *Varroa* mites, actin and α tubulin were used as housekeeping gene references.

The QuantiGene assay was performed according to the manufacturer’s instructions (Affymetrix, Inc., User Manual, 2010) with the addition of a heat denaturation step prior to hybridization of the sample with the oligonucleotide probes. Samples in a 20 μL volume were mixed with 5 μl of the supplied probe set in the well of a PCR microplate, followed by heating for 5 minutes at 95°C using a thermocycler. Heat-treated samples were maintained at 46°C until use. The 25 μl samples were transferred to an Affymetrix hybridization plate for overnight hybridization. Before removing the plate from the thermocycler, 75 μl of the hybridization buffer containing the remaining components were added to each sample well. The PCR microplate was then removed from the thermocycler; the content of each well (~100 μl) was then transferred to the corresponding well of a Hybridization Plate (Affymetrix) for overnight hybridization. After signal amplification, median fluorescence intensity (MFI) for each sample was captured on a Luminex 200 machine (Luminex Corporation).

### *Varroa* counts

Bees were collected in 70% alcohol solution and shaken for 10 minutes on a Burrel (Model 75) Wrist Action Shaker. Bottles were emptied onto a VWR 1/8 inch US Standard Testing Sieve (Cat # 57334–242) to collect the *Varroa* shaken off the bees. Washed *Varroa* fell through the sieve onto a weigh boat, and the sieve, with the bees on top, was shaken by hand to collect any *Varroa* mites that had not washed off immediately. The bottle was checked for any *Varroa* that had not poured out onto the sieve. *Varroa* mites were then counted. To determine the number of bees in each bottle, 10 bees from each bottle were weighed and average bee weight was calculated. Weight of all bees was then divided by the average bee weight to calculate number of bees. The *Varroa* count was divided by number of bees and multiplied by 100 to determine number of *Varroa*/100 bees.

### Statistical methods

Differences among sites for bee number at study initiation was tested using one-way analysis of variance with site as the sole fixed effect. Mean separation was performed using Fisher’s Protected LSD. A logistic regression was used to predict the probability of hive survival (AGM score >1) given bee counts. The number of bees associated with a collapsed hive at the first observation of collapse was considered as the bee count for collapsed hives. For non-collapsed hives, the minimum bee count across the 4 measurement periods was used as the bee count for the hive. The logistic regression modeled the binomial response of collapsed or live hives as a function of bee number, site, and the interaction between site and bee number. The relationship was not found to differ by site and the across site model is reported here.

Viruses were considered present if the Quantigene Plex 2.0 value was above the background level. Percent prevalence was calculated as the number of hives with the virus over the number of hives that were sampled. As the study progressed, the number of sampled hives decreased due to hive loss.

Pearson’s Product-Moment Correlation analysis was performed to test for linear relationships between viral loads in *Varroa* and bees for DWV and IAPV. A logarithmic transformation was applied to the Quantigene units data before conducting the correlation analysis.

The relationship between *Varroa* number and viral load was examined using a repeated measures analysis of covariance. The viral load (log transformed) was fitted as a function of *Varroa* number and collection time, while modeling the repeated measures on hives across time with an autoregressive covariance structure of order 1. The analysis was conducted by location and virus. The same analysis was performed for the relationship between viral load and *Nosema* load.

Hives that had an AGM score of ≤1 at any time during the study were considered to be collapsed. Data from the first 3 collection periods was used in a repeated measures analysis that fitted individual viral loads or *Varroa* load as a function of the binary variable of collapsed or live hives, collection time, and the interaction between hive status and collection time. The data from the April collection period was not used since it was unknown if the hives collapsed at a subsequent time period and viral data from collapsed hives was limited at the final collection period. The analysis was conducted by location and virus.

### Predictive modeling of colony losses indicates that combination of *Varroa* destructor and replication of DWV are the major markers of colony losses

A multi-factor model to predict colony losses was developed by first selecting a subset of predictor variables from the profile of nine viruses, *Varroa* mite counts, *Nosema* load, 3-, 7-, 10-, and 14-day moving averages for minimum and maximum temperatures (defined as extreme weather conditions), and average minimum and average maximum temperature. For collapsed hives, the virus profile, *Nosema* load, and *Varroa* counts at the time of collapse or at the previous collection period were used in the model and the weather variables for the time period between the time of collapse and the previous time period were used. For live hives, the profile at the February collection period and the weather variables between the January and February collection periods were used. The February profiles were selected for live hives as they represented the time period when most colony losses occurred. The subset of variables included in the final modeling process was selected by consensus of variable importance ranking by Random Forest [53] and LASSO regression techniques [54]. Stepwise logistic regression was applied to the subset of variables to develop a final model.

Results are considered significant at P<0.05. The Random Forest and LASSO regressions were performed in R [55,56]. All other statistical analyses were conducted using SAS/STAT software.

## Supporting Information

S1 Table Virus prevalence by location and time period for all colonies and for the subset of colonies with measurements at all time periods. Virus prevalence was determined at all sites for all live colonies at each sampled time point and compared to survived hives at the end of the trial (same colonies over time). N represents number of hives at each analysis.
(DOCX)

S2 Table Least-squares means for relative level and replication of DWV and IAPV in survived hives versus collapsed hives at each site. P-values are reported for effect of hive status at the first three measurement periods.
(DOCX)

S3 Table Bee counts in survived hives at each site. Bee counts were calculated by applying the Indicounter (WSC Regexperts) software on bee frame images.
(DOCX)
