# Supplementary material for: Prospective Large-Scale Field Study Generates Predictive Model Identifying Major Contributors to Colony Losses
Source: PLoS Pathog. 2015 Apr 13;11(4):e1004816. doi: 10.1371/journal.ppat.1004816 (PMC4395366; doi:10.1371/journal.ppat.1004816)
Supplement: S1 Table — Virus prevalence was determined at all sites for all live colonies at each sampled time point and compared to survived hives at the end of the trial (same colonies over time). N represents number of hives at each analysis. (DOCX) [file ppat.1004816.s001.docx]

**Table S1:** Virus prevalence by location and time period for all colonies and for the subset of colonies with measurements at all time periods.

|  |  | All Available Colonies | | | | | Same colonies over time | | | | |
| --- | --- | --- | --- | --- | --- | --- | --- | --- | --- | --- | --- |
| Virus | Time | Site 1 | Site 2 | Site 3 | All Sites | N | Site 1 | Site 2 | Site 3 | All Sites | N |
| ABPV | October | 16.9 | 35.2 | 24.6 | 25.3 | 170 | 17.9 | 50.0 | 21.4 | 24.1 | 79 |
| ABPV | January | 54.7 | 44.7 | 76.8 | 59.6 | 156 | 56.4 | 58.3 | 75.0 | 63.3 | 79 |
| ABPV | February | 2.0 | 39.6 | 65.8 | 32.8 | 137 | 2.6 | 41.7 | 64.3 | 30.4 | 79 |
| ABPV | April | 86.0 | 89.5 | 93.5 | 89.2 | 93 | 87.2 | 91.7 | 92.9 | 89.9 | 79 |
| BQCV | October | 23.7 | 61.1 | 49.1 | 44.1 | 170 | 20.5 | 75.0 | 50.0 | 39.2 | 79 |
| BQCV | January | 77.4 | 61.7 | 41.1 | 59.6 | 156 | 79.5 | 83.3 | 35.7 | 64.6 | 79 |
| BQCV | February | 0.0 | 37.5 | 52.6 | 27.7 | 137 | 0.0 | 41.7 | 50.0 | 24.1 | 79 |
| BQCV | April | 79.1 | 47.4 | 83.9 | 74.2 | 93 | 79.5 | 58.3 | 82.1 | 77.2 | 79 |
| CBPV | October | 49.2 | 88.9 | 78.9 | 71.8 | 170 | 46.2 | 91.7 | 82.1 | 65.8 | 79 |
| CBPV | January | 96.2 | 74.5 | 85.7 | 85.9 | 156 | 94.9 | 83.3 | 85.7 | 89.9 | 79 |
| CBPV | February | 0.0 | 45.8 | 92.1 | 41.6 | 137 | 0.0 | 58.3 | 92.9 | 41.8 | 79 |
| CBPV | April | 86.0 | 42.1 | 80.6 | 75.3 | 93 | 84.6 | 58.3 | 78.6 | 78.5 | 79 |
| DWV | October | 98.3 | 96.3 | 86.0 | 93.5 | 170 | 97.4 | 91.7 | 96.4 | 96.2 | 79 |
| DWV | January | 94.3 | 93.6 | 69.6 | 85.3 | 156 | 92.3 | 91.7 | 60.7 | 81.0 | 79 |
| DWV | February | 66.7 | 97.9 | 57.9 | 75.2 | 137 | 59.0 | 100.0 | 64.3 | 67.1 | 79 |
| DWV | April | 76.7 | 100.0 | 83.9 | 83.9 | 93 | 79.5 | 100.0 | 82.1 | 83.5 | 79 |
| IAPV | October | 10.2 | 42.6 | 33.3 | 28.2 | 170 | 10.3 | 50.0 | 32.1 | 24.1 | 79 |
| IAPV | January | 60.4 | 51.1 | 17.9 | 42.3 | 156 | 69.2 | 58.3 | 10.7 | 46.8 | 79 |
| IAPV | February | 0.0 | 37.5 | 44.7 | 25.5 | 137 | 0.0 | 33.3 | 50.0 | 22.8 | 79 |
| IAPV | April | 72.1 | 94.7 | 74.2 | 77.4 | 93 | 74.4 | 91.7 | 71.4 | 75.9 | 79 |
| KBV | October | 6.8 | 16.7 | 12.3 | 11.8 | 170 | 2.6 | 16.7 | 14.3 | 8.9 | 79 |
| KBV | January | 34.0 | 31.9 | 33.9 | 33.3 | 156 | 38.5 | 33.3 | 32.1 | 35.4 | 79 |
| KBV | February | 0.0 | 20.8 | 36.8 | 17.5 | 137 | 0.0 | 16.7 | 39.3 | 16.5 | 79 |
| KBV | April | 65.1 | 47.4 | 83.9 | 67.7 | 93 | 64.1 | 58.3 | 85.7 | 70.9 | 79 |
| LSV | October | 82.1 | 98.0 | 100.0 | 93.2 | 162 | 78.6 | 91.7 | 100.0 | 87.5 | 56 |
| LSV | January | 100.0 | 92.7 | 100.0 | 97.6 | 164 | 100.0 | 91.7 | 100.0 | 98.2 | 56 |
| LSV | February | 82.9 | 97.9 | 100.0 | 93.7 | 127 | 82.1 | 100.0 | 100.0 | 91.1 | 56 |
| LSV | April | 100.0 | 93.8 | 100.0 | 98.5 | 67 | 100.0 | 100.0 | 100.0 | 100.0 | 56 |
| VDV | October | 64.4 | 96.3 | 77.2 | 78.8 | 170 | 56.4 | 100.0 | 75.0 | 69.6 | 79 |
| VDV | January | 92.5 | 85.1 | 75.0 | 84.0 | 156 | 92.3 | 100.0 | 67.9 | 84.8 | 79 |
| VDV | February | 13.7 | 81.3 | 89.5 | 58.4 | 137 | 15.4 | 83.3 | 89.3 | 51.9 | 79 |
| VDV | April | 97.7 | 100.0 | 93.5 | 96.8 | 93 | 97.4 | 100.0 | 92.9 | 96.2 | 79 |
