# Supplementary material for: Prospective Large-Scale Field Study Generates Predictive Model Identifying Major Contributors to Colony Losses
Source: PLoS Pathog. 2015 Apr 13;11(4):e1004816. doi: 10.1371/journal.ppat.1004816 (PMC4395366; doi:10.1371/journal.ppat.1004816)
Supplement: S2 Table — P-values are reported for effect of hive status at the first three measurement periods. (DOCX) [file ppat.1004816.s002.docx]

**Table S2:** Least-squares means for relative level and replication of DWV and IAPV in survived hives versus collapsed hives at each site.

|  |  | Hive | Least-squares means (Log10 QG) | | | P-value for survived vs collapsed | | |
| --- | --- | --- | --- | --- | --- | --- | --- | --- |
| Site | Virus | Status | October | January | February | October | January | February |
| 1 | DWV | Survived | 2.72 | 2.48 | 1.38 | 0.249 | 0.386 | 0.330 |
|  |  | Collapsed | 3.19 | 2.86 | 1.86 |  |  |  |
|  | Replication of DWV | Survived | 0.46 | 0.91 | 0.32 | 0.005 | 0.805 | 0.412 |
|  |  | Collapsed | 1.01 | 0.96 | 0.51 |  |  |  |
|  | IAPV | Survived | 0.07 | 0.72 | 0.00 | 0.763 | 0.147 | 1.000 |
|  |  | Collapsed | 0.03 | 0.54 | 0.00 |  |  |  |
|  | Replication of IAPV | Survived | 0.02 | 0.56 | 0.02 | 0.869 | 0.744 | 0.864 |
|  |  | Collapsed | 0.00 | 0.52 | 0.00 |  |  |  |
| 2 | DWV | Survived | 1.74 | 1.58 | 2.58 | 0.623 | 0.112 | 0.002 |
|  |  | Collapsed | 1.93 | 2.22 | 3.78 |  |  |  |
|  | Replication of DWV | Survived | 0.37 | 0.37 | 0.17 | 0.821 | 0.198 | <.0001 |
|  |  | Collapsed | 0.42 | 0.66 | 1.31 |  |  |  |
|  | IAPV | Survived | 0.29 | 0.75 | 0.25 | 0.888 | 0.355 | 0.011 |
|  |  | Collapsed | 0.26 | 0.49 | 0.93 |  |  |  |
|  | Replication of IAPV | Survived | 0.34 | 0.60 | 0.09 | 0.481 | 0.187 | 0.136 |
|  |  | Collapsed | 0.24 | 0.40 | 0.30 |  |  |  |
| 3 | DWV | Survived | 1.61 | 0.88 | 1.02 | 0.887 | 0.002 | 0.340 |
|  |  | Collapsed | 1.55 | 2.06 | 0.42 |  |  |  |
|  | Replication of DWV | Survived | 0.42 | 0.24 | 0.56 | 0.509 | 0.163 | 0.203 |
|  |  | Collapsed | 0.53 | 0.48 | 0.18 |  |  |  |
|  | IAPV | Survived | 0.34 | 0.06 | 0.60 | 0.997 | 0.049 | 0.097 |
|  |  | Collapsed | 0.33 | 0.45 | 0.03 |  |  |  |
|  | Replication of IAPV | Survived | 0.29 | 0.10 | 0.61 | 0.269 | 0.963 | 0.021 |
|  |  | Collapsed | 0.14 | 0.11 | 0.06 |  |  |  |
