# Supplementary material for: Prospective Large-Scale Field Study Generates Predictive Model Identifying Major Contributors to Colony Losses
Source: PLoS Pathog. 2015 Apr 13;11(4):e1004816. doi: 10.1371/journal.ppat.1004816 (PMC4395366; doi:10.1371/journal.ppat.1004816)
Supplement: S3 Table — Bee counts were calculated by applying the Indicounter (WSC Regexperts) software on bee frame images. (DOCX) [file ppat.1004816.s003.docx]

| **Site** | **Number of Bees/ Hive** | | | | **% bees from start point** | | | |
| --- | --- | --- | --- | --- | --- | --- | --- | --- |
|  | **October** | **Jan** | **Feb** | **April** | **October** | **Jan** | **Feb** | **April** |
| Site 1 | 11491 | 5426 | 5591 | 20498 | 100% | 47% | 49% | 178% |
| Site 2 | 11853 | 11710 | 10366 | 6280 | 100% | 99% | 87% | 53% |
| Site3 | 8087 | 5870 | 4462 | 12949 | 100% | 73% | 55% | 160% |
